# Superconductor to resistive state switching by multiple fluctuation events in NbTiN nanostrips

**DOI:** 10.1038/s41598-019-42736-3

**Published:** 2019-05-29

**Authors:** M. Ejrnaes, D. Salvoni, L. Parlato, D. Massarotti, R. Caruso, F. Tafuri, X. Y. Yang, L. X. You, Z. Wang, G. P. Pepe, R. Cristiano

**Affiliations:** 1 Consiglio Nazionale delle Ricerche – Institute of Superconductors, Innovative Materials and Devices, Via Campi Flegrei, 34, 80078 Pozzuoli NA, Italy; 2 Dipartimento di Fisica, Università degli Studi di Napoli ‘Federico II’, I-80126 Napoli, Italy; 3 Consiglio Nazionale delle Ricerche – Institute of Superconductors, Innovative Materials and Devices, c/o Complesso di Monte S. Angelo, via Cinthia, 80126 Napoli, Italy; 4 Dipartimento di Ingegneria Elettrica e delle Tecnologie dell’Informazione, Università degli Studi di Napoli ‘Federico II’, I-80125 Napoli, Italy; 5 State Key Lab of Functional Materials for Informatics, Shanghai Institute of Microsystem and Information Technology (SIMIT), Chinese Academy of Sciences (CAS), 865 Changning Rd., Shanghai, 200050 P.R. China; 6 CAS Center for Excellence in Superconducting Electronics (CENSE), 865 Changning Rd., Shanghai, 200050 P.R. China

**Keywords:** Superconducting properties and materials, Superconducting devices, Superconducting devices

## Abstract

We report on measurements of the switching current distributions on two-dimensional superconducting NbTiN strips that are 5 nm thick and 80 nm wide. We observe that the width of the switching current distributions has a non-monotonous temperature dependence, where it is constant at the lowest temperatures up to about 1.5 K, after which it increases with temperature until 2.2 K. Above 2.5 K any increase in temperature decreases the distribution width which at 4.0 K is smaller than half the width observed at 0.3 K. By using a careful analysis of the higher order moments of the switching distribution, we show that this temperature dependence is caused by switching due to multiple fluctuations. We also find that the onset of switching by multiple events causes the current dependence of the switching rate to develop a characteristic deviation from a pure exponential increase, that becomes more pronounced at higher temperatures, due to the inclusion of higher order terms.

## Introduction

One of the fundamental questions in the physics of low dimensional superconducting strips is the nature of the intrinsic mechanism that is responsible for the passage from the superconducting to the resistive state. In a deterministic description, the switching process occurs when the current in the strip reaches the maximum value (called the critical current) determined by the maximum velocity of the Cooper pairs. Above this velocity, superconductive pairing is no longer possible and the strip undergoes to a resistive switching. In practice, fluctuation effects of the order parameter known as phase slips can induce stochastic premature switching^1–3^. Phase slip phenomena are receiving great attention in literature both for one-dimensional (1D) and two-dimensional (2D) systems. The interest for 1D superconducting strips or nanowires is due to the fact that, for instance, they can undergo quantum phase slip that is topological quantum fluctuations of the superconducting order-parameter field through which tunneling occurs between current-carrying states^2–7^. Recently, the regime when fluctuations induced switching processes are due to multiple phase slip (MPS) events has emerged as observed in 1D wires^2,3,8–10^.

The recent usage of 2D superconducting strips as superconducting single photon detectors (SSPDs)^11^ has contributed to the interest into the physics of phase slip phenomena in these strips. Dark counts, i.e. the false events in a detector, are very low in SSPDs and constitute one of the most attractive figures-of-merit. Murphy *et al*.^12^ has recently investigated “[…] quantum, thermal and multiple phase slips as generators of dark counts” in quasi-2D NbN strips.

In this work, we investigate phase slip events in 2D NbTiN strips, a material of great interest and widely used in SSPD applications, where it was originally introduced because it lowered the dark count rate^13^ and has recently reached a noise equivalent power at the 10^−20^ W/Hz^1/2^ level^14^. We measure the switching current distributions in a wide interval of temperatures from 4.2 K down to 0.3 K. The standard deviations of the switching distributions show an extended region of temperatures where MPS event switching occurs. We further analyze the measured data and find that it is the same fluctuation that causes switching by MPS events, as the ones that cause switching by single phase slip events. Finally, we quantify the energy scale of the fluctuation phenomenon. Beyond the interest from the fundamental point of view for the similarity of the physics involved in our and in 1D strips, our result may have also consequences for the diagnostic of SSPD operation mode. It is worthwhile to remember that when the switching distribution width is small then the region of bias currents affected by spontaneous switching is also small. In other words, from the point of view of dark counts, it is preferable operate the SSPD where the switching distribution width is small. Contrary to what happens in the case of NbN SSPD where the smallest switching distribution width values occur below 1.5 K^12^, in our case the smallest switching distribution width occurs at the highest temperature where the multi-phase slip process is dominating. For clarity, in the following a quantized phase change of 2π in the order parameter will be considered a phase slip event. Hence, escape through an energy saddle point which does not involve a vortex core^15–17^, vortex-antivortex pairs splitting^18,19^ and single vortices crossing an edge barrier^18,20–22^ are possible origins of phase slip events.

## Experiment

**Figure 1:**
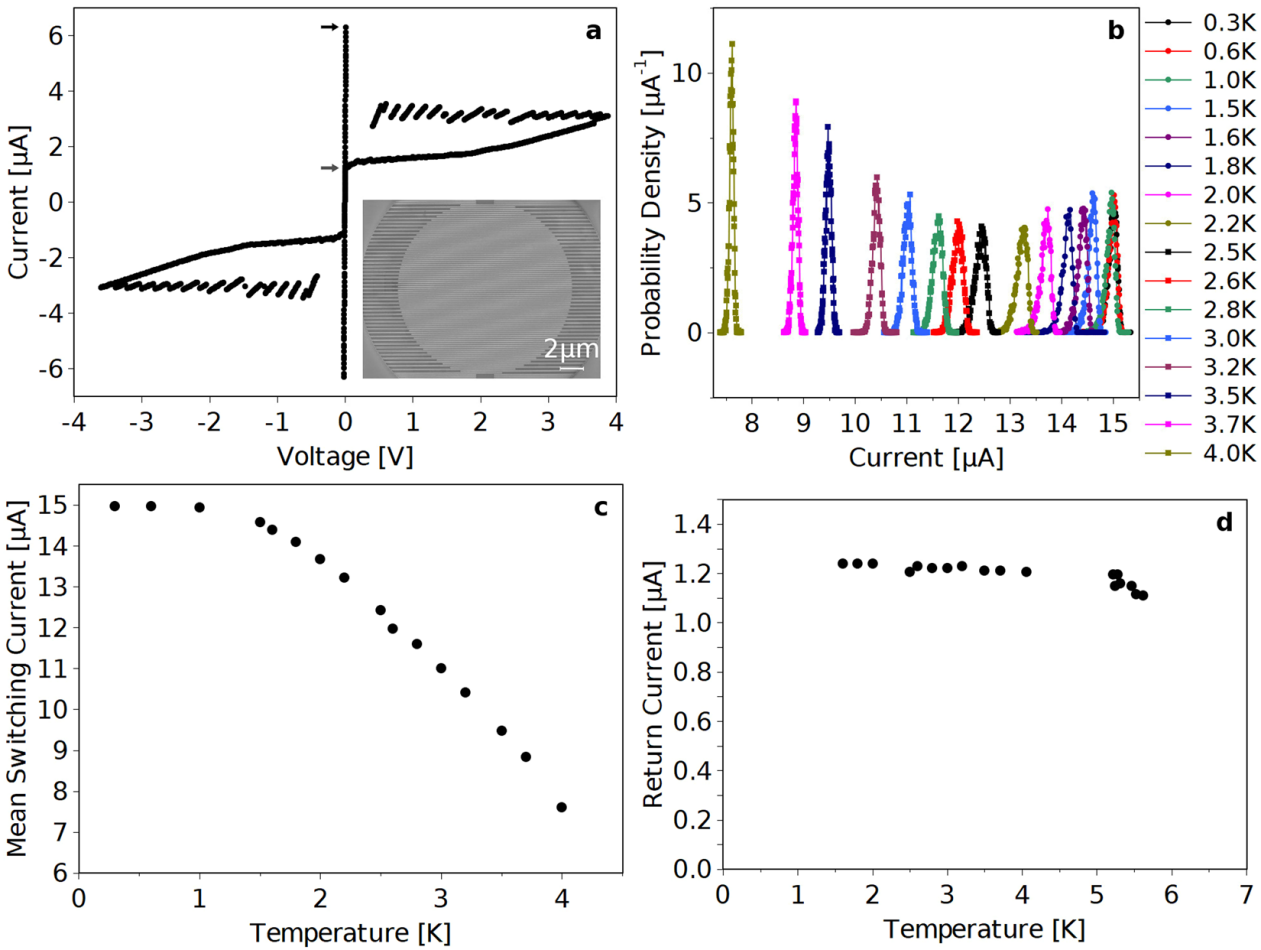
Measured data from the NbTiN strip. (**a**) Measured current-voltage characteristic at 4.2 K. The switching current is indicated by the upper black arrow whereas the re-trapping current is indicated by the lower grey arrow. Inset: Scanning Electron Micrograph of the device. (**b**) Measured switching-current distributions for temperatures between 0.3 K (right-most) and 4 K (left-most). (**c**) Measured temperature dependence of the mean switching current. (**d**) Measured temperature dependence of the re-trapping current.

We measured NbTiN meandered nanostrips, 5 nm thick (d) and 80 nm wide (w). The superconducting order parameter in our strip is in the 2-dimensional regime, because the coherence length of our ultrathin NbTiN films is much lower than the strip width whereas it is similar to the thickness. The NbTiN thin films were deposited on double-side polished thermally oxidized Si substrates by reactive DC-magnetron sputtering in an Ar+ N_2_ gas mixture at room temperature. The films were patterned into a nanostrip structure by electron-beam lithography, reactive-ion etching (RIE). The area of the meandered nanostrips is round with a diameter of 15 μm (see inset in Fig. 1a). Detailed fabrication process can be found in ref.^23^.

Measurements were performed anchoring the samples to the ^3^He pot chamber of HELIOX Oxford cryostat, whose cryogenic and electronic characteristics (thermal anchoring and various stages of filtering) together with details on low noise electronics used for the experiment are reported elsewhere^24,25^. The critical temperature of our strip is 6.6 K. A typical current-voltage characteristic measured at 4.2 K is shown in Fig. 1a. As the bias current is swept from zero to higher values, the strip exhibits an abrupt transition from the superconducting state (zero voltage) to a finite voltage state at the switching current, *I*_*SW*_. The strip will stay in the finite voltage state until the current is reduced below the re-trapping current at which point the strip becomes superconducting again. This gives rise to a hysteretic current-voltage characteristic. When we subjected our NbTiN strip to repeated current bias sweeps, we observed that *I*_*SW*_ stochastically varies from sweep to sweep, as has been observed in many different superconductive systems at low temperatures. To further investigate the physics of switching in NbTiN strips, we measured the distribution of switching currents, *P*(*I*_*SW*_), at different temperatures. Each *P*(*I*_*SW*_) was obtained using a current sweep rate of 845 µA/s and included 10000 switching events^24,25^.

### Analysis

In Fig. 1b we show the measured *P*(*I*_*SW*_)s for all the measured temperatures and we note a peculiar non-monotonic temperature dependence. Roughly speaking, it is seen that the *P*(*I*_*SW*_)s initially become broader when the temperature is increased from the 0.3 K, then the trend inverts and the distributions become increasingly narrower with increasing temperature (i.e. stronger fluctuations). The former part of this trend is intuitive and has been seen in many different coherent systems^3,12,26–28^, where it typically is attributed to thermal excitation across an energy barrier of a single physical process. On the contrary, the latter part of the trend is counter-intuitive. Nonetheless, similar behaviors have been observed in Josephson junctions^25,29,30^, Al point contact^10^ and in 1D superconducting strips^2,3,8,9^ where it is attributed to the onset of MPS event switching. We find it interesting to observe a similar behavior in 2D NbTiN strips and our subsequent analysis shows that the resistive switching in the higher temperature region is caused by thermal activation of MPS events. In order to give a comprehensive insight into the device in Fig. 1c we show the temperature dependence of the mean switching current of the measured distributions in Fig. 1b and in Fig. 1d we show the measured temperature dependence of the re-trapping current.

**Figure 2:**
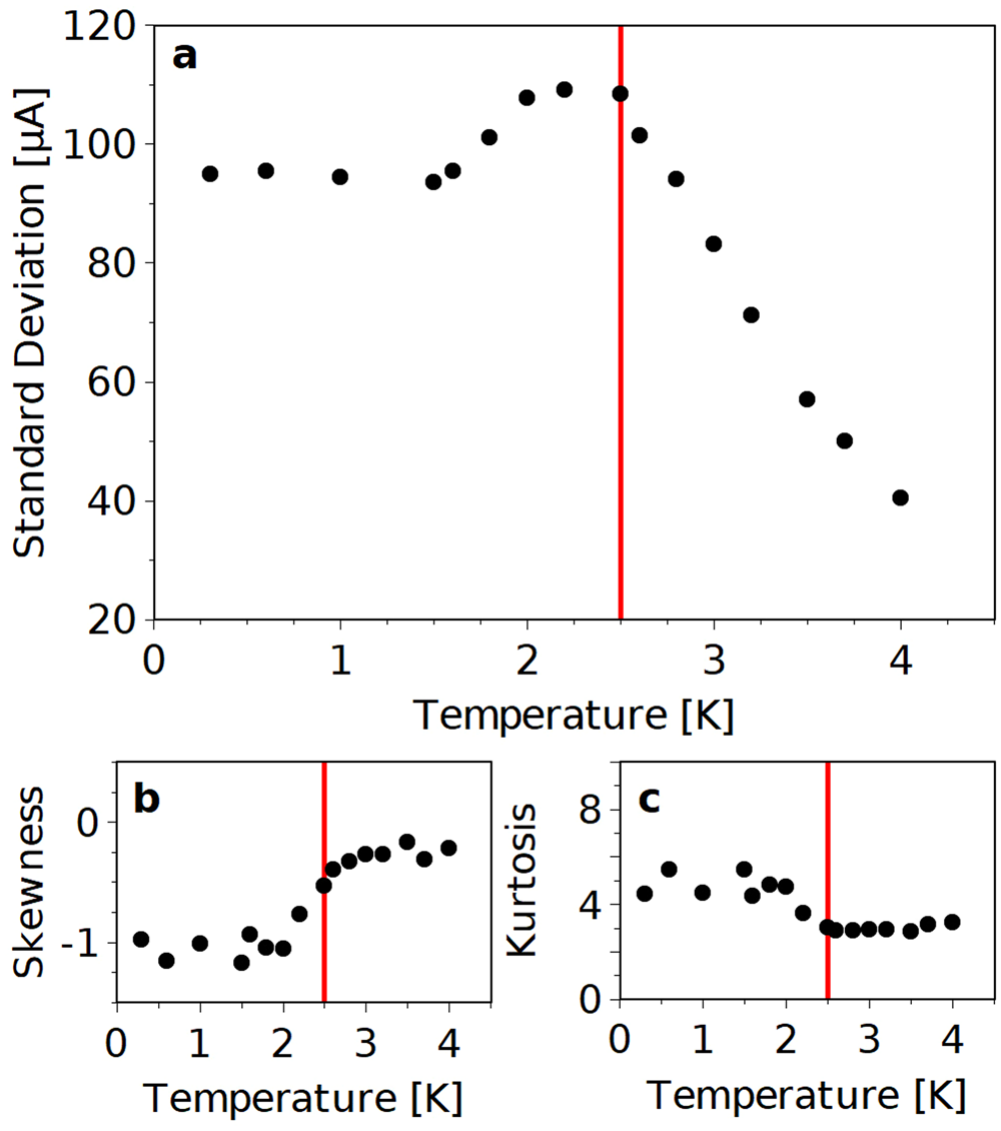
Analysis of the measured switching current distributions of Fig. 1b. (**a**) Standard deviations as a function of temperature. (**b**) The skewness as a function of temperature. (**c**) The kurtosis as a function of temperature. For reference the red line is shown to indicate the change from single event switching to MPS switching, that occurs around 2.5 K.

We start by calculating the standard deviation, σ, of the measured *P*(*I*_*SW*_)s for each temperature and the results are shown in Fig. 2a where the two regimes described above are distinctly visible. In detail, σ is constant in the temperature range from 0.3 K to 1.5 K after which it increases with temperature in the range from 1.5 K to 2.2 K and finally it exhibits a prominent decrease with increasing temperature from 2.5 K to 4.0 K and this decrease is the main result of this paper. We note that the σ at 4.0 K is 42% of the value measured at 0.3 K. The measured dependence of σ at temperatures below 2.5 K is very similar to what has been observed in 2D NbN strips, where it was attributed to switching due to quantum tunnelling and thermally excited phase slips^12,31^. As mentioned above, the decrease of σ measured above 2.5 K has been observed in 1D superconducting strips where this trend is attributed to switching by MPS. In this regime the single physical event is not by itself energetically sufficient to properly trigger a full superconductor to resistive state transition, which instead only happens when several events occur as also predicted by numerical simulation^2,8,9^. Hence, the observed switching events are due to the occurrence of multiple coincident events. In this way the counterintuitive observation is explained; even though individual thermal fluctuations are more frequent at higher temperatures, the necessity of having multiple events to induce resistive switching reduces σ. At higher temperatures there are several circumstances that reduce the impact of a single fluctuation event. Firstly, the switching current decreases causing a lower dissipation during the phase slip event. Secondly, both the electron and phonon heat capacities increase reducing the impact of the dissipation on the electron temperature during the phase slip event.

In order to further investigate if the decrease in σ is due to MPS event switching for our 2D NbTiN strips, we calculate the skewness, *S*, from the measured switching distributions as:

1 $$S={{\rm{\Sigma }}}_{i=1}^{N}\,{p}_{i}\frac{{({I}_{SW,i}-\langle {I}_{SW}\rangle )}^{3}}{{\sigma }^{3}}$$

Where *N* is the number of bins in the switching distribution, *p*_*i*_ is the probability of switching in bin *i* and *I*_*SW,i*_ is the switching current of bin *i*. In Fig. 2b we show the results for all temperatures and it is seen that the skewness is about −1 for temperatures below 2.2 K and about −0.2 for temperatures above 2.5 K. This is further evidence for a switching caused by a single physical event below 2.2 K and MPS events at temperatures above 2.5 K as the former case should give a skewness of −1 and the latter a skewness of about zero^4,8^. In detail, since an Arrhenius-type activation formula for the escape rate^32^ provides a characteristic asymmetric tail on the ascending side of the switching distributions, the skewness tends to −1^4^. When the bias current dependence of the escape rate deviates from an Arrhenius-type behaviour due to MPS event switching, the histograms are more symmetric and the skewness tends progressively to zero^33^.

Likewise, we have calculated the kurtosis, *K*, for the measured switching distributions as:

2 $$K={{\rm{\Sigma }}}_{i=1}^{N}\,{p}_{i}\frac{{({I}_{SW,i}-\langle {I}_{SW}\rangle )}^{4}}{{\sigma }^{4}}$$

In Fig. 2c we show the results for all temperatures and it is seen that the kurtosis is between 4.5 and 5.5 for temperatures below 2.2 K and about 3 for temperatures above 2.5 K. Thus again proving that the switching is caused by a single physical event below 2.2 K^4,8^ and MPS events at temperatures above 2.5 K.

**Figure 3:**
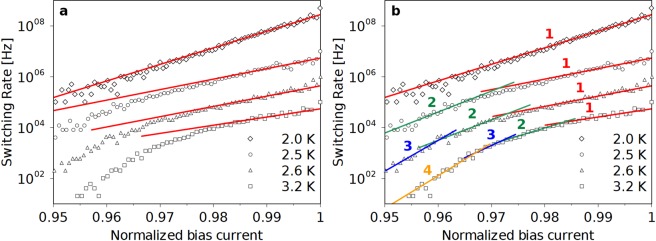
Analysis of the influence of MPS events on the switching rates from the superconducting state to the resistive state obtained from the measured distributions of Fig. 1b. (**a**) Switching rates for four different temperatures. The solid lines are fits to the data at high bias current obtained using Eq. 7 without the MPS contribution. (**b**) Switching rates for four different temperatures as in (**a**). The solid lines are fits to the data using Eq. 7 with the indicated higher order MPS events included. For clarity in both (**a**) and (**b**) we have multiplied both the data and fits by a factor of 10 (2.6 K), 100 (2.5 K) and 5000 (2.0 K) respectively.

To gain more insight into the physics of the switching of our NbTiN strips we calculate the rate of switching, *Γ*, as a function of current for each switching distribution in the following way^34^:

3 $$\Gamma ({I}_{i})=\frac{\nu }{{\rm{\Delta }}I}\,\mathrm{ln}(\frac{{\sum }_{j=i}^{N}{p}_{j}}{{\sum }_{j=i+1}^{N}{p}_{j}})$$

where *ν* is the current sweep rate and *ΔI* is the current bin size that for our data is between 4 nA and 8 nA depending on the width of the switching histogram. In Fig. 3a we show *Γ* for four different temperatures where we have normalized the bias current to the maximum measured current in the *P*(*I*_*SW*_), i.e. *I*_N_. It is seen that *Γ* has an exponential increase with bias current in the temperature range below 2.5 K whereas for higher temperatures *Γ* develops a characteristic drop at low bias currents. Furthermore, the drop becomes more pronounced as the temperature is increased. At this point, it is clear that for NbTiN strips at intermediate temperatures the switching distributions from the superconducting state to the resistive state are made narrow by the necessity of MPS events for switching at low currents. This reduces the observed dark count rates for NbTiN-based SNSPDs as was originally reported in ref.^13^. It is also important to note that, while operating an SNSPD in the MPS event switching region, the thermal fluctuations in the detector are much more frequent than the measured dark count rate.

In 1D superconducting nanowires the characteristic drop of *Γ* seen in Fig. 3a has been modelled as a progressive increase of the number of events necessary for the MPS event to cause the resistive switching^8,9^. In this physical picture the fluctuation mechanism is the same in both the region of MPS event switching and thermally activated single event switching. Inspired by this, we have further investigated if the MPS event switching in our 2D NbTiN nanostrip can be attributed to a single physical process. We have chosen to do this using a statistical approach because the 1D model^8,9^ is not applicable to our 2D system. We assume that the *Γ* that we observe in the region of MPS event switching is due to the coincidence of several thermally activated fluctuation events which by themselves occur with a rate, *Γ*_F_, that is given by^32^:

4 $${{\Gamma }}_{F}({I}_{B},T)={{\rm{\Omega }}}_{F}({I}_{B},T){e}^{-\frac{{\rm{\Delta }}F({I}_{B},T)}{{k}_{B}T}}$$

where *Ω*_F_ is the attempt frequency, *ΔF* is the energy barrier for the fluctuation process and *I*_B_ is the bias current normalized to the critical current. We also assume that the energy barrier shape is smooth around the critical current and that *Ω*_F_ does not depend on bias current close to the critical current. In this way the role of the bias current is to control *ΔF* and the approach is compatible with several different phase slip mechanisms^18–22^. Thus we can series develop *ΔF* in *I*_B_ to first order around *I*_B_ = 1 obtaining for a fixed temperature:

5 $${{\Gamma }}_{F}({I}_{B})=K{e}^{\alpha {I}_{B}}$$

here $K={\rm{\Omega }}\,\exp (-\frac{{\rm{\Delta }}{\rm{F}}(1)+{\rm{\Delta }}{\rm{F}}^{\prime} (1)}{{{\rm{k}}}_{{\rm{B}}}{\rm{T}}})$, ${\rm{\alpha }}={\rm{\Delta }}{\rm{F}}^{\prime} (1)/{{\rm{k}}}_{{\rm{B}}}{\rm{T}}$ and ΔF′(I_B_) = dΔF(I_B_)/dI_B_. Since we consider each individual fluctuation event independent, their statistics is Poissonian and we can calculate the occurrence rate of *n* coincident fluctuation events as:

6 $${\Gamma }_{n}({I}_{B})=\frac{{({\rm{\Delta }}t{p}_{S})}^{n-1}}{(n-1)!}{[{{\Gamma }}_{F}({I}_{B})]}^{n}$$

where *Δt* is the time window of coincidence between events, *p*_*S*_ is the probability of spatial coincidence and we assume that ${\Gamma }_{F}({I}_{i})\,\Delta t\,{p}_{S}\ll 1$. In this notation we have *Γ*_1_ = *Γ*_*F*_. Inserting Eq. 5 into Eq. 6 we obtain:

7 $${\Gamma }_{n}({I}_{i})={A}_{n}{e}^{n\alpha {I}_{B}}$$

where *A*_*n*_ = *K*^*n*^ (*Δt p*_*S*_)^*n*−1^/(*n*−1)!. As shown in Fig. 3a we can fit the experimental data at lower temperatures with Eq. 7 using only *n* = 1. However, at 2.5 K we cannot fit *Γ* by a single exponential, but a good agreement is obtained by including the n = 2 term of Eq. 7. As the temperature increases it is clearly seen that more terms of higher order are needed to properly fit the data (see Fig. 3b). In detail, for each temperature we proceed by fitting *Γ* in the high bias current region with Eq. 7 using *n* = 1, and we obtain *α* and *A*_1_. Subsequently, we iteratively fit the switching rates at lower bias current with the *n*-th order formula, using only *A*_*n*_ as a fitting parameter while *α* is not changed. As shown in Fig. 3b, there is a good correspondence between the fit and the data for the three different temperatures in the MPS event switching regime. We believe that, this analysis with a fixed *α* proves that the MPS event switching in our 2D NbTiN nanostrip is caused by a single physical process^35^. In order to estimate the characteristic energy scale of this physical process, we have examined the *Γ* measured at 2.0 K. At this temperature *Γ* is dominated by single events of thermal activation across an energy barrier which we analyse in the framework of de-pairing of vortex-antivortex pairs (VAP). The expected count rate is given by^18^:

8 $${\Gamma }_{VAP}(I)={\Omega }_{VAP}\exp (-\frac{A(T)}{\varepsilon \,{k}_{B}\,T}[\mathrm{ln}(\frac{2.6}{{I}_{B}})-1+\frac{{I}_{B}}{2.6}])$$

where *Ω*_*VAP*_ is the attempt frequency, *A(T)* is the vortex interaction parameter, ε is the averaged polarizability of a VAP within the entire VAP population and *I*_*B*_ is the normalized bias current. The resulting fitting parameter *A(T)/ε* is 47 meV, which is about one order of magnitude lower than what was observed in similar nanostrips based on NbN^36^.

## Conclusions

We have shown that the resistive switching distributions of NbTiN strips have a pronounced region at high temperatures where the switching is caused by MPS events. In this temperature region the width of the switching distribution is reduced down to values below the ones observed at the lowest temperature. The third and fourth moments of the switching distribution were also investigated and confirmed the switching origin to be of MPS event type at high temperatures. We furthermore found that the bias current dependence of the switching rate develops a characteristic deviation from a pure exponential increase caused by MPS events of the same type. The deviation was found to accommodate regions of higher order MPS event switching as the temperature increases. Since the switching rate is reduced when it originates in MPS events, these results could explain why SNSPDs based on NbTiN have shown lower dark count rates^13,14^.

## Supplementary information

Supporting Information

## Data Availability

the authors will make the data available to who is interested.
