# Author Correction: Superconductor to resistive state switching by multiple fluctuation events in NbTiN nanostrips

**DOI:** 10.1038/s41598-019-56346-6

**Published:** 2019-12-20

**Authors:** M. Ejrnaes, D. Salvoni, L. Parlato, D. Massarotti, R. Caruso, F. Tafuri, X. Y. Yang, L. X. You, Z. Wang, G. P. Pepe, R. Cristiano

**Affiliations:** 1 Consiglio Nazionale delle Ricerche – Institute of Superconductors, Innovative Materials and Devices, Via Campi Flegrei, 34, 80078 Pozzuoli, NA Italy; 2 Dipartimento di Fisica, Università degli Studi di Napoli ‘Federico II’, I-80126 Napoli, Italy; 3 Consiglio Nazionale delle Ricerche – Institute of Superconductors, Innovative Materials and Devices, c/o Complesso di Monte S. Angelo, via Cinthia, 80126 Napoli, Italy; 4 Dipartimento di Ingegneria Elettrica e delle Tecnologie dell’Informazione, Università degli Studi di Napoli ‘Federico II’, I-80125 Napoli, Italy; 5 State Key Lab of Functional Materials for Informatics, Shanghai Institute of Microsystem and Information Technology (SIMIT), Chinese Academy of Sciences (CAS), 865 Changning Rd., Shanghai, 200050 P.R. China; 6 CAS Center for Excellence in Superconducting Electronics (CENSE), 865 Changning Rd., Shanghai, 200050 P.R. China

Correction to: *Scientific Reports* 10.1038/s41598-019-42736-3, published online 29 May 2019

This Article contains an error in Figure 2A, where the Y-axis is incorrectly labelled.

“Standard Deviation [µA]”

should read:

“Standard Deviation [nA]”

The correct Figure 2 appears below as Figure 1.

**Figure 1:**
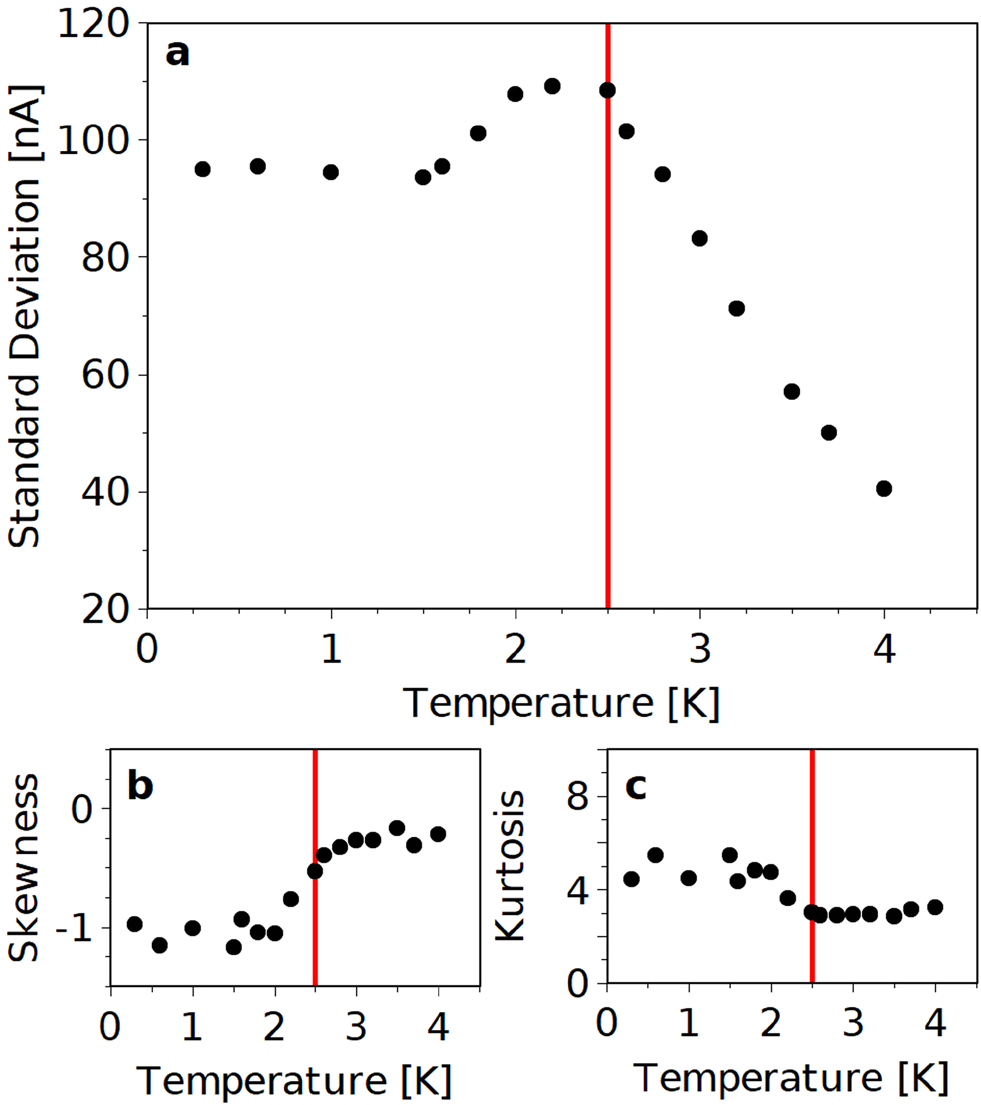
.

