# Supplementary material for: Superconductor to resistive state switching by multiple fluctuation events in NbTiN nanostrips
Source: Sci Rep. 2019 May 29;9:8053. doi: 10.1038/s41598-019-42736-3 (PMC6541640; doi:10.1038/s41598-019-42736-3)
Supplement: Supplementary file 1 — Supporting Information [file 41598_2019_42736_MOESM1_ESM.pdf]

Supporting Information

Superconductor to resistive state switching by multiple fluctuation events in NbTiN nanostrips

M. Ejrnaes[1], D. Salvoni[2,1], L. Parlato[2,1], D. Massarotti[3,1], R. Caruso[2,1], F. Tafuri[2,1], X. Y. Yang[4], L. You[4,5], Z. Wang[4,5] , G. P. Pepe[2,1], R. Cristiano[1]

[1] Consiglio Nazionale delle Ricerche – Institute of Superconductors, Innovative Materials and Devices, Via Campi Flegrei 34, I-80078 Pozzuoli, Italy

[2] Dipartimento di Fisica, Università degli Studi di Napoli ‘Federico II’, I-80125 Napoli, Italy

[3] Dipartimento di Ingegneria Elettrica e delle Tecnologie dell'Informazione, Università degli Studi di Napoli ‘Federico II’, I-80125 Napoli, Italy

[4] State Key Lab of Functional Materials for Informatics, Shanghai Institute of Microsystem and Information Technology (SIMIT), Chinese Academy of Sciences (CAS), 865 Changning Rd., Shanghai, 200050, P. R. China.

[5] CAS Center for Excellence in Superconducting Electronics (CENSE), 865 Changning Rd., Shanghai, 200050, P. R. China.

We have performed preliminary measurements of a nominally identical NbTiN device realized in a separate fabrication run. The device had a critical current of 12.5 µA at 4.2 K as can be seen in the current voltage characteristic shown in supplementary figure S1a. At 1.5 K and 6.0 K we measured the switching distributions, as described in the main article, by subjecting the NbTiN strip to 10000 bias current sweeps and recording the switching current for each sweep. From the distribution of switching current we calculate the bias current dependence of the switching rate using equation 3 of the main article and the results are shown in supplementary figure S1b. It is seen that at the temperature of 1.5 K the switching rate has an exponential dependence on the bias current indicating that it is probably in the single event switching region like the device presented in the manuscript at this temperature. Instead, at 6.0 K the characteristic drop in switching rate at low bias currents is observed demonstrating that a change to MPS event switching has occurred.

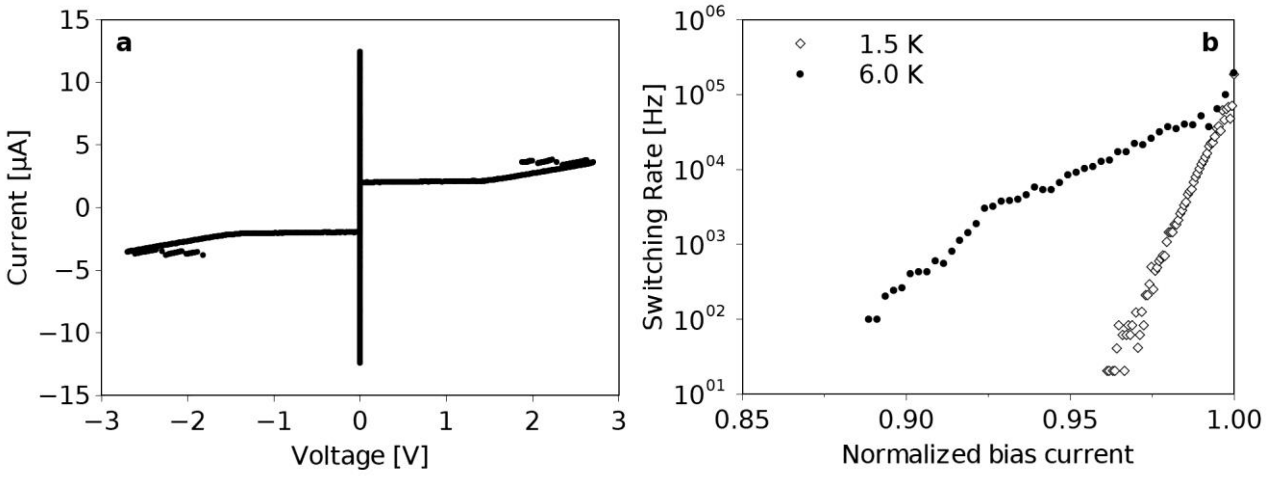

Supplementary Figure S1. Measurement results for the second NbTiN strip. (**a**) Current voltage characteristic at 4.2 K. (**b**) Switching rate as a function of bias current measured at 1.5 K (◊) and 6.0 K (●).
